# Evaluation of the salivary level of glutathione reductase, catalase and free thiol in patients with oral lichen planus

**DOI:** 10.1186/s12903-023-03242-1

**Published:** 2023-08-09

**Authors:** Fahimeh Rezazadeh, Dorsa Mahdavi, Nima Fassihi, Hossein Sedarat, Elham Tayebi Khorami, Amir Tabesh

**Affiliations:** 1 Dept. of Oral and Maxillofacial Medicine, School of Dentistry, Shiraz University of Medical Sciences, Shiraz, Iran; 2 Student research committee, School of Dentistry, Shiraz University of Medical Sciences, Shiraz, Iran; 3 Undergraduate medical Student, Jahrom University of Medical Sciences, Jahrom, Iran

**Keywords:** Oral lichen planus, Saliva, Catalase, Glutathione reductase, Free-thiol

## Abstract

**Objective:**

Oral lichen planus (OLP) is a usual chronic inflammatory disease of the oral mucosa with malignant capacity, whose pathogenesis is not yet well known. Free radicals and reactive oxygen species may have a vital role in the pathogenesis of oral lichen planus. This study aimed to assess Glutathione reductase, catalase, and free thiol levels in the saliva of OLP patients and compare it with healthy people.

**Methods:**

In this cross-sectional study, 35 patients with OLP and 20 healthy people were involved. Five mL of whole, unstimulated saliva samples were collected in the morning, and the salivary levels of antioxidants were measured by ELISA technique. In this experiment, sex, age and OLP types were also evaluated.

**Results:**

There was a significant decrease in the patients’ salivary level of Glutathione reductase (0.2043 mU/ml in patients and 0.3901 mU/ml in the control group) and catalase (0.1525 mU/ml in patients and 0.2700 mU/ml in the control group) (p = 0.001). But there were no differences between the two groups regarding free-thiol levels (0.0586 mU/ml in patients and 0.0569 mU/ml in the control group) (p = 0.7). However, there was no correlation between age and gender with the antioxidants’ contents. There was a significant decrease in glutathione reductase and catalase in the erosive type than in the non-erosive type.

**Conclusions:**

In this study, we found that the salivary levels of Glutathione reductase and Catalase were lower in OLP patients than in the healthy group, which means these antioxidants were affected by OLP and also associated with the type of it. So salivary Glutathione reductase and Catalase levels may be used as biomarkers for OLP monitoring and treatment.

## Introduction

Oral Lichen planus (OLP) has been described as a chronic mucocutaneous inflammatory illness, in which oral mucosa is usually involved [1]. It is more predominant in middle-aged women and white patients [1, 2]. The occurrence of OLP is 1–2% in the overall population, while its dominance in the Asian population is 2.6% [3].

Clinical manifestations of OLP include erythema and white lines on the oral mucosa, described as Wickham striae, with various appearances, such as papular, reticular, atrophic, plaque, erosive, and bullous [4, 5]. Erosive or atrophic types of OLP can trigger symptoms varying from a burning sensation to severe pain that influence the patients’ condition of life [4]. The particular etiology of OLP stays uncertain. Collected data verify the participation of cell-mediated immune dysfunction in the progress of OLP [4, 6]. Keratinocyte apoptosis and lymphocytic infiltration have been surveyed to likely stimulate the creation of a cell-mediated immune reaction [7]. Lichen planus is assumed to result from an atypical T-cell-mediated immune reaction in which basal epithelial cells are distinguished as exotic due to alterations in the antigenicity of their cell surface. The reason for this immune-mediated basal cell injury is undiscovered. Also, it is yet undiscovered whether lichen planus characterizes a solitary disease procedure or numerous intently associated entities with related clinical demonstrations [8, 9].

Catalase is an ordinary enzyme discovered in almost all living creatures exposed to oxygen (such as plants, bacteria and animals). It catalyzes the breakdown of hydrogen peroxide into water and oxygen [10]. Hydrogen peroxide is a destructive product of many routine metabolic processes; to avoid damage to cells and tissues, it should be quickly transformed into other, less dangerous materials [11].

Glutathione (GSH) is important in preserving suitable tasks and avoiding oxidative stress in human cells. Furthermore, it acts as the main character in the metabolism and clearance of xenobiotics, performs as a cofactor in confident detoxifying enzymes, contributes to transportation, and regenerates antioxidants such as Vitamins C and E into their reactive forms [12]. The GSR gene in humans encodes the enzyme glutathione reductase (GR), also referred to as glutathione-disulfide reductase (GSR). The conversion of glutathione disulfide (GSSG) to glutathione sulfhydryl form (GSH) is catalyzed by glutathione reductase. The ratio of GSSG/GSH present in the cell is an essential feature in correctly preserving the oxidative equilibrium of the cell; it is substantial that the cell maintains high levels of reduced glutathione and a low level of oxidized glutathione disulfide. This narrow balance is kept by glutathione reductase, which catalyzes the reduction of GSSG to GSH [12].

Apart from the character that disulfides act in protein construction, alterations of reactive cysteine thiols can change the role of proteins and can be included in the modulation of enzymatic activity. Thiols thus function not only in normal cell signaling via S-nitrosation, S-glutathionylation, or S-sulfonation but can also be permanently oxidized by aging and illness and disturb protein tasks [13, 14]. In the existence of oxidants (thiol oxidase), protein thiols can also develop mixed disulfides with glutathione (GSH) [15]. Thiol clusters are essential supporters of the antioxidant team and have been shown to terminate ROS and other free radicals through enzymatic and non-enzymatic methods [16]. Total thiol groups of proteins are primarily responsible for their antioxidant response and may behave as a sensitive marker of oxidative stress [17].

Total thiol groups are highly vulnerable to oxidation and known as one of the most significant plasma sacrificial antioxidants. Once the cells are exposed to oxidative stress, thiol groups are the first antioxidants that are affected. Thiol oxidase is an enzyme that catalyzes the chemical reaction between the reduced type of thiol (free thiol) to the oxidized type of thiol (disulfide form) [17].

The use of saliva as a diagnostic fluid is a moderately topical development. Saliva is a mottled oral fluid arising from major and minor salivary glands. Oral fluid, frequently described as the reflection of the body’s condition, is a precise method to be surveyed for disease and health observation. Saliva is the first line of defense against oxidative stress (OS), the primary source of several systemic and oral illnesses. Significant causes of oral free radicals and reactive oxygen species (ROS) are periodontitis, tobacco smoke and other oral diseases. Saliva contains antioxidants: albumin, ascorbate, uric acid and enzymes that establish the antioxidant capacity of saliva [18].

The inequity in antioxidants is considered crucial in this illness [19, 20]. Increased lipid peroxidation levels, oxidative stress, and serum or salivary oxygen free radicals precede inflammatory procedures and harm the cellular membranes [21]. Reduced antioxidant protection and elevated oxidative stress can play a significant part in the etiology of several diseases, including diabetes [22], Periodontitis [23], Psoriasis [24], Vitiligo [25], and digestive system complaints [20].

Regarding previous studies and since no similar studies evaluated these biological markers, we aimed to determine the salivary levels of catalase, glutathione reductase and thiol oxidate in patients with OLP and the association between these values and the types of disease.

## Methods and materials

In this cross-sectional study (during 2019–2020), 35 OLP patients, who were referred to the oral and maxillofacial disease department of the Shiraz dental faculty, were enrolled. Existence of oral lichen planus was confirmed by a specialist based on clinical or histopathologic evaluations. Among those who were referred to Shiraz dental faculty’s oral and maxillofacial disease department, 20 healthy individuals were selected as the control group (age and sex matched). Data, including the severity of the disease (type of OLP), were collected for all patients. The participants were inquired about general health situations, medicine intake and smoking addiction. The exclusion criteria involved the following: other local and systemic inflammatory illnesses, the use of anti-inflammatory drugs and analgesics, the use of vitamin C and E 2 months before the study, smoking, which raises the levels of oxidants, and already being under cure for OLP.

Subjects were requested to restrain from chewing gum, using lipstick, eating or drinking any liquids except water for two hours prior to sampling saliva. They washed their mouth for 30 s with clean water, and the water was eliminated. Subjects were trained not to attempt to produce saliva, talk, or think about foods. Five mL of whole, unstimulated saliva samples were collected in a sterile container and frozen at -20 c until analysis afterward.

Salivary levels of antioxidants were measured by ELISA (Enzyme-Linked Immunosorbent Assay) technique using a Kiazist kit (Kiazist, Iran). Data were analyzed by SPSS version 25.0 (SPSS Inc., IL, USA), applying mean ± SD and frequency (%). Independent t-test and chi-square were used to compare all data, such as age, sex, and types of lichen planus, between groups. A repeated measure ANOVA (RM-ANOVA) was used to assess the changes in some measurements during times. P < 0.05 was significant.

## Result

In this study, 35 OLP patients, including fifteen males and twenty females, and 20 healthy individuals, including nine males and eleven females, were examined. With regard to the Chi-square test, both groups were similar in sex distribution (p = 0.87). The mean age of patients was 33.46 ± 13.41, and the mean age of healthy people was 31.85 ± 5.98; thus, there was no significant difference between the mean ages of the two groups (p = 0.54).

Based on clinical examinations, among the OLP patients, we had 13 with the erosive type (37.1%), and 22 with the non-erosive type (62.9%).

Statistical data analyses clarified that the mean Glutathione reductase was 0.2043(± 0.1769) mU/ml in OLP patients and 0.3901 (± 0.1060) mU/ml in the control group. The mean saliva level of catalase was 0.1525 (± 0.0618) mU/ml in OLP patients and 0.2700 (± 0.0563) mU/ml in the control group. The salivary level of free thiol was 0.0586 (± 0.0162) mU/ml in OLP patients and 0.0569 (± 0.0155) mU/ml in the control group.

According to Independent T-test, there was a significant decrease in Glutathione reductase and catalase in the OLP group (p = 0.001) and there were no differences in free-thiol between the two groups (p = 0.7).

Based on the One-Way ANOVA test, there was no association between Glutathione reductase, Ctalase, and Free thiol concerning age and sex. The main difference between the two groups was the distinct levels of Glutathione reductase and Catalase (Table 1). There was a significant decrease in glutathione reductase and catalase in the erosive group (Table 2).

**Table 1:** Comparison of group, age and sex in different antioxidants

		P-value
Glutathion reductase	**Group** **Age** **Sex**	0.002 0.255 0.408
Catalase	**Group** **Age** **Sex**	0.000 0.542 0.562
Free thiol	**Group** **Age** **Sex**	0.505 0.301 0.251

**Table 2:** Comparison of different antioxidants in erosive and non-erosive group

	Type	Number	Mean	St.Deviation	P-value
glutathion_reductase (mU/ml)	**erosive** **Non-erosive**	13 22	0.9369 0.2696	0.1849 0.7416	0.000
Catalase (mU/ml)	**erosive** **Non-erosive**	13 22	0.1023 0.1822	0.1374 0.5999	0.000
free_thiol (mU/ml)	**erosive** **Non-erosive**	13 22	0.5540 0.6053	0.1496 0.1704	0.375

## Discussion

At present, it has been declared that the difference in stages of free radicals and ROS with antioxidants can play a significant part in the onset of numerous inflammatory illnesses [26].

In this research, the relationship between the mean salivary level of some antioxidants (Glutathione reductase, Catalase and Free thiol) and OLP was analyzed. Based on the present results, there was a significant reduction in glutathione reductase and catalase salivary levels in OLP patients compared to healthy participants due to the type of OLP. However, various researchers assessed other antioxidants level in OLP patients, and we compared them with our study. In accordance with the present study, Sedigheh Bakhtiari et al. manifested OLP was correlated with a reduction in Uric Acid (UA) stages in the saliva. They presented salivary UA as a biomarker that could be utilized for observing and treating OLP [27].

Corroborating the findings of our study, Tugba Tunali-Akbay et al. demonstrated that total antioxidant capacity (TAC) and GSH accumulation were lower in patients with OLP [28]. In addition to our results, Daniela Miricescu et al. illustrated that OS reduced the antioxidant levels in the oral cavity. Uric acid, albumin and TAC are significant and hopeful salivary biomarkers for checking the oral OS. Salivary TAC actions were significantly lower in periodontitis, OLP and smoker patients than in the control groups, indicating an essential oxidative procedure in the oral cavity [18].

Shirzaiy M et al. concluded that levels of Gluthatione peroxidase and superoxide dismutase were remarkably lower in OLP patients compared healthy people [29].

In another study, Sezer E et al. found significantly lower erythrocyte catalase levels in a group of patients with OLP [30].

Atena shiva et al. clarified that OLP groups have more significant cellular lipid peroxidation compared to healthy controls and low stage of (total antioxidant activity) TAA than controls. Patients with OLP are assumed to be further in danger of antioxidant-oxidative stress inequality [31].

In contrast to this study, Suma et al. explained in their examination of the saliva of brain tumor patients that salivary protein thiols were significantly increased [32]. While the patients’ conditions were different than ours, perhaps with the reduction of free thiol in the reduced form of intracellular glutathione, there was another compensating pathway with a different form of extracellular free thiol protein, and the final measurable free thiol remained constant in our results.

Goes Gonzaga et al. found that level of GSH in OLP patients was not remarkably different in comparison with healthy people. However, despite of conducted researches on the effectiveness of oxidative stress markers of saliva in OLP patients, he proposed a better standardization of methods for more accurate conclusion [33].

According to our study, age and gender are not associated with antioxidants level. So the level of antioxidants is not affected by the age and gender of the patients.

Similar to these findings, Rezazadeh et al. showed no significant connection between the level of salivary IL-10 and sex and age in oral lichen planus [34].

As we realized in this research, glutathione reductase and catalase significantly declined in the erosive type of OLP compared to the non-erosive type. Azizi et al. also displayed that salivary and plasma stages of total antioxidant grade in erosive OLP patients were lower than in the healthy participants [35].

The limitation of this study was detecting an approved laboratory to achieve reliable saliva test results. Different labs and several kits were estimated, and the reliability and validity of the one used in our study were verified. Another limitation of this study was the small size of the sample.

## Conclusion

Present results showed a significant decrease in Glutathione reductase and Catalase salivary levels in OLP patients compared to the control group. There was also a correlation between the type of OLP and antioxidant levels; Glutathione reductase and Catalase salivary levels were lower in erosive than non-erosive type; we also found no correlation between age and gender with these antioxidant contents. Our findings show these antioxidants were affected by OLP and also related to its type. Hence, salivary Glutathione reductase and Catalase levels may be used as biomarkers for OLP monitoring and treatment.

## Data Availability

The datasets during the current study are not publicly available due to confidentiality of the patients’ data, but they will be available upon editorial reasonable request through the corresponding author contact details(email): etayebi@sums.ac.ir.
